# Electrophysiological Evidence for Impaired Central Pain Modulation in Parkinson's Disease

**DOI:** 10.1002/mds.70004

**Published:** 2025-08-23

**Authors:** Dilara Kersebaum, Josephine Lassen, Julia Forstenpointner, Manon Sendel, Sophie‐Charlotte Fabig, Sonja Nölker, Juliane Sachau, Steffen Paschen, Daniela Berg, Ralf Baron, Philipp Hüllemann

**Affiliations:** ^1^ Division of Neurological Pain Research and Therapy, Department of Neurology University Hospital Schleswig‐Holstein, Campus Kiel Kiel Germany; ^2^ Department of Neurology University Hospital Schleswig‐Holstein, Campus Kiel Kiel Germany; ^3^ Department of Neurology and Neurorehabilitation MEDICLIN Klinikum Soltau Soltau Germany

**Keywords:** central pain modulation, laser‐evoked potential habituation, pain, pain mechanisms, Parkinson's disease

## Abstract

**Background:**

There is a remarkable overlap between structures involved in pain perception and the pathophysiology of Parkinson's disease (PD). Recent efforts to allocate pain into mechanistic subtypes require a better understanding of central pain processing in PD patients.

**Objectives:**

The aim of this study was to show electrophysiological evidence for altered central pain processing in a patient group with PD, taking their reported pain, somatosensory profile, and motor symptoms as well as pharmacotherapy into account.

**Methods:**

The laser‐evoked‐potential (LEP)‐habituation paradigm and quantitative sensory testing were applied to PD patients (n = 41) in the off‐l‐dopamine (levodopa) state. The development of LEP amplitudes and laser pain ratings over the course of 100 painful stimuli was compared to those of an age‐matched control group (n = 24). The Unified Parkinson's Disease Rating Scale (UPDRS) III and the painDETECT questionnaire and medical history, including pharmacotherapy, were assessed and analyzed in context with LEP and pain habituation aiming to find an electrophysiological proxy for central sensitization.

**Results:**

Patients exhibited a significantly reduced capacity for LEP habituation regardless of clinically reported pain and sensory profile. No association of EEG data has been found with the mean l‐DOPA equivalent dose taken by the patients.

**Conclusions:**

We hereby report electrophysiological evidence for an impaired central pain modulation in PD patients regardless of pain presentation and individual sensation. Further exploration of abnormal central pain processing in PD using methods like the LEP habituation paradigm or conditioned pain modulation protocol is needed in larger cohorts. © 2025 The Author(s). *Movement Disorders* published by Wiley Periodicals LLC on behalf of International Parkinson and Movement Disorder Society.

## Graphical abstract

Our study demonstrates electrophysiological evidence of impaired central pain modulation in Parkinson's disease (PD) patients using laser‐evoked potential habituation and quantitative sensory testing. PD patients exhibited reduced habituation to painful stimuli, independent of reported pain or medication.

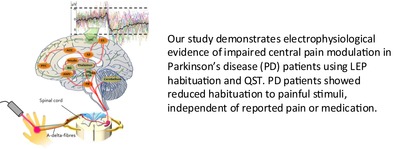

## Introduction

**FIG. 1:**
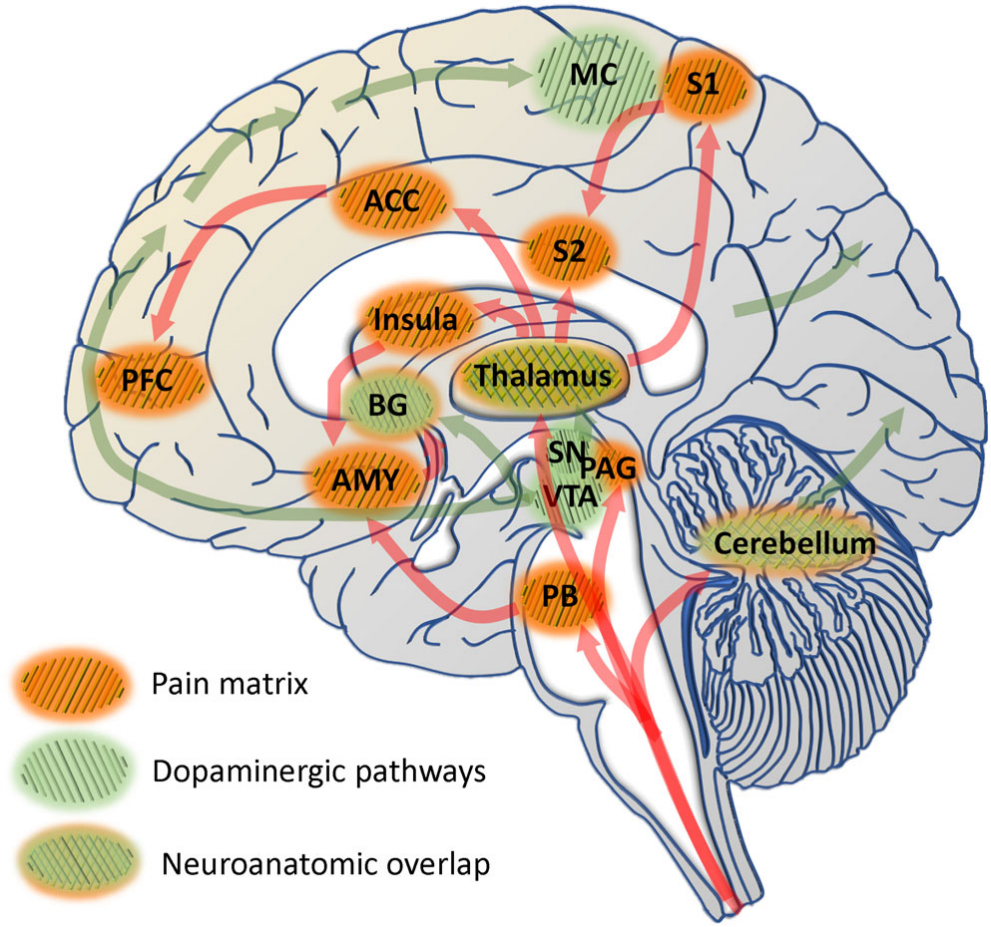
Neuroanatomic overlap: pain matrix and dopaminergic pathways. Cortical areas: ACC, anterior cingulate cortex; AMY, amygdala; BG, basal ganglia; MC, motor; PAG, periaqueductal gray; PB, parabrachial nucleus; PFC, prefrontal; S1, primary somatosensory; S2, secondary somatosensory; SN, substantia nigra; VTA, ventral tegmental area.

In 1817 James Parkinson described the symptom pain in his *Essay on a Shaking Palsy*.^1^ Since then, we have come to understand that in Parkinson's disease (PD), this nonmotor symptom can be (1) an early symptom, (2) caused by various mechanisms, and (3) appears under fluctuations.^2^, ^3^, ^4^, ^5^, ^6^, ^7^ Although its negative impact on quality of life is evident and some patients consider it their major troublesome symptom,^8^, ^9^ pain still fails to get the clinical recognition it deserves.^10^, ^11^, ^12^ The challenge behind pain is perhaps its collection of “many syndromes under one umbrella.”^2^ Until today, there have been several efforts to classify pain in PD^2^, ^13^, ^14^, ^15^, ^16^, ^17^ (for recent review, see Tinazzi and colleagues^18^), and we recognize a remarkable overlap between structures involved in pain perception/processing on the one hand and PD pathology on the other (Fig. 1, adapted from Bushnell and colleagues and Aarsland and colleagues^19^, ^20^).

Consequently, disturbed central pain processing in PD patients comes naturally to mind. The interest in pain mechanisms transcends the academic realm and is key to a targeted treatment with higher probability of therapeutic success.^21^, ^22^, ^23^, ^24^ Still, among the mechanisms discussed to be involved in PD‐related pain, changes in central pain modulation and central sensitization (CS) are of increasing interest.^7^, ^9^, ^25^, ^26^, ^27^ CS as defined by the International Association for the Study of Pain (“Increased responsiveness of nociceptive neurons in the central nervous system to their normal or subthreshold afferent input”^28^) cannot be directly measured in humans and is therefore assessed through proxies (eg, questionnaires, sensory signs and symptoms, conditioned pain modulation [CPM], and habituation paradigms^29^, ^30^, ^31^, ^32^, ^33^, ^34^, ^35^). Laser‐evoked potentials (LEP) reflective of A‐delta and C‐fiber function as well as the spinothalamic tract and cortical structures (eg, insular and anterior cingulate cortex [ACC]^36^, ^37^, ^38^, ^39^, ^40^, ^41^, ^42^, ^43^) are “the easiest and most reliable of the neurophysiological methods for assessing function of nociceptive pathways.”^44^ Previous threshold and LEP studies pointed toward the involvement of central structures in PD‐related pain.^6^, ^27^, ^45^, ^46^, ^47^, ^48^ The LEP‐habituation paradigm examining the central adaptation to repetitive painful stimuli through the diminution of the N2P2 amplitudes and laser pain ratings (LPR) over the course of time is considered to be an indicator of CS processes in several chronic pain conditions.^34^, ^35^, ^49^, ^50^, ^51^, ^52^, ^53^

Due to the call for an improved understanding of pain‐driving mechanisms and central pain processing in PD and the need for distinctive diagnostic tools in clinical practice,^4^, ^7^, ^27^, ^45^ we have posed the following research questions:

1. Is there any evidence for disturbed central pain processing in off‐state PD patients that can be assessed through the LEP‐habituation paradigm?

If yes,

2 Is there any connection between reported pain or typical sensory signs and symptoms of CS with the habituation capacity?
3 Are there any sensory findings that may be indicative of disturbed central pain processing in PD patients?
4 Do results of routine PD‐related tests allow conclusions about the state of central pain processing?
5 Is there an impact of PD‐specific pharmacotherapy (ie, equivalent dosage of l‐DOPA (levodopa) per day or agonists) on central pain processing (habituation effect)?

## Patients and Methods

For a detailed description of the methodology, including general information and inclusion and exclusion criteria, see Data S1: Methods.

### Study Cohort and Design

PD patients with and without pain were recruited. LEP data laser perception threshold, laser pain (LP) threshold, LEP amplitudes, and N2 and P2 latency, and LPRs of 24 age‐matched controls were included. All patients were in an off‐state during the conduction of electrophysiological and neurophysiological tests. The assessment of the somatosensory system of PD patients in the off‐state is a particular strength of this study and was ethically possible as patients were recruited from the Department of Neurology, University Hospital Schleswig‐Holstein Campus Kiel (UKSH) where they presented electively for assessing their eligibility for deep brain stimulation (DBS), thus needing to be off medication for clinical purposes (ie, prior to the Unified Parkinson's Disease Rating Scale [UPDRS]). Our patients were in the Hoehn & Yahr stages 2 and 3.^54^, ^55^, ^56^

The study was approved by the Ethics Committee of the UKSH (study protocol number: D532/16), complied with the Declaration of Helsinki, and was registered at the German Clinical Trials Register (DRKS00011207). All patients gave their written informed consent.

### Assessed Parameters

We assessed the painDETECT questionnaire (PDQ),^57^, ^58^, ^59^, ^60^, ^61^, ^62^, ^63^, ^64^, ^65^ quantitative sensory testing (QST) according to the German Research Network of Neuropathic Pain,^55^, ^56^ LEPs,^25^, ^35^, ^48^, ^51^, ^66^, ^67^, ^68^, ^69^, ^70^ and the UPDRS III.^71^ LEP recording and documentation of pain ratings as well as the LEP‐habituation paradigm were conducted according to previously published protocols.^51^, ^66^, ^67^ For the LEP‐habituation protocol, we used an Nd:YAP 1340 Stimul Laser (neodymium:yttrium‐aluminiumperovskite, DEKA Lasertechnologie GmbH, Mainburg, Germany) with a beam diameter of 5 mm and a stimulus duration of 5 ms. A total number of 100 stimuli were applied in four blocks at 25 stimuli, with 8 to 12 ms between each stimulus. The laser stimulator was moved slightly within the dermatome of the testing area to avoid receptor fatigue.

### Statistical Analysis

A detailed description of the methodology is shown in Data S1: Methods.

Comparisons have been made in terms of LEP and LP habituation (analysis of variance [ANOVA], see later) as well as group variables (Mann‐Whitney *U* test, MWU, see later).

To account for research questions 2 to 5, patients were divided into the following subgroups:

- Clinical presentation with pain (PDQ)
- Loss of small‐fiber function, loss of large‐fiber function, and CS‐typical signs and symptoms (QST)
- Routine clinical PD test (patients with a UPDRS score >42.3 vs. <42.3, with 42.3 being the mean score)
- Pharmacotherapy

Descriptive statistics (mean, standard deviation, 95% confidence interval) and inferential statistical methods (ANOVA, Mann‐Whitney *U* test) were used to examine differences in the habituation of LEP and LP responses and other parameters between Parkinson's subgroups and healthy controls. Additionally, correlations between habituation‐related electroencephalogram (EEG) parameters and clinical variables (eg, pain, QST, UPDRS, medication) were calculated.

## Data Sharing

All data are available upon reasonable request to the corresponding author.

## Results

In total, 41 PD patients (15 women, 26 men) were recruited, and 24 age‐matched healthy individuals (10 women, 14 men) were included. EEG data of 30 patients complied with our inclusion criteria. Two patients were not able to follow the instructions during off‐state. For 9 patients, EEG data were not eligible for analysis due to artifacts, an unfavorable signal‐to‐noise ratio, or fatigue effects, resulting in overlap with α‐wave EEG, making the detection of the LEPs impossible within all four consecutive simulation blocks.

For descriptive statistics of the controls and the entire patient group, including *P*‐values for the comparison of group variables, see Table 1. Table 2 presents a comparison of the assessed parameters divided by patients with current pain ≥1 versus 0. Table S1 presents the pharmacotherapy of each patient.

**TABLE 1:** Demographic data, EEG, and pain values for controls and all PD patients

	Controls	Patients	*P*‐value
Age (y)	58.8 ± 6.8 [56;61.7]	61.8 ± 9.8 [58.7;64.9]	ns
Disease duration (y)	na	9 ± 4.4 [7.6;10.4]	na
Height (cm)	175.1 ± 9.2 [171.2;179]	173.2 ± 9.7 [170.1;176.3]	ns
L‐DT	0.9 ± 0.3 [0.8;1]	1.2 ± 0.5 [1.1;1.4]	0.003
L‐PT	2.1 ± 0.4 [1.9;2.3]	2.4 ± 0.6 [2.2;2.6]	0.04
N2P2‐1 (μV)	14 ± 7 [11;17]	11.7 ± 8.4 [8.5;14.8]	ns
N2P2‐2 (μV)	11.7 ± 5.9 [9.1;14.2]	10.9 ± 8.9 [7.6;14.2]	ns
N2P2‐3 (μV)	9.7 ± 5.5 [7.3;12]	10.5 ± 11.4 [6.3;14.8]	ns
N2P2‐4 (μV)	8 ± 5 [5.9;10.1]	10.4 ± 8.8 [7.1;13.7]	ns
LEP‐HQ	0.6 ± 0.3 [0.5;0.7]	1 ± 0.6 [0.8;1.2]	0.003
Pain‐1 (NRS)	3.6 ± 1.2 [3.1;4.2]	4.9 ± 2.2 [4.2;5.7]	0.025
Pain‐2 (NRS)	3 ± 1.2 [2.5;3.5]	4.1 ± 1.9 [3.5;4.7]	0.009
Pain‐3 (NRS)	2.7 ± 1.1 [2.3;3.2]	4.1 ± 1.9 [3.5;4.7]	0.002
Pain‐4 (NRS)	2.6 ± 1.2 [2.1;3.1]	3.7 ± 1.9 [3.1;4.3]	0.025
LP‐HQ	0.7 ± 0.3 [0.6;0.9]	0.8 ± 0.3 [0.7;0.9]	ns
N2‐1 (ms)	239 ± 15 [232.6;245.3]	243.9 ± 29.2 [232.8;255]	ns
N2‐2 (ms)	235.7 ± 15.5 [229.1;242.2]	246.8 ± 31.6 [234.8;258.9]	ns
N2‐3 (ms)	238.3 ± 16.6 [231.3;245.3]	244 ± 34.7 [230.8;257.2]	ns
N2‐4 (ms)	234 ± 19.2 [225.9;242.1]	243.2 ± 30.8 [231.5;254.9]	ns
P2‐1 (ms)	333.5 ± 26.1 [322.4;344.5]	328.7 ± 40.5 [313.3;344.1]	ns
P2‐2 (ms)	335 ± 34.9 [320.3;349.8]	321.2 ± 38.2 [306.6;335.7]	ns
P2‐3 (ms)	335.9 ± 28 [324.1;347.7]	328.1 ± 39.2 [313.2;343]	ns
P2‐4 (ms)	333.3 ± 34.4 [318.8;347.9]	327.6 ± 36.1 [313.8;341.3]	ns
PDQ score	na	6.1 ± 6.8 [3.8;8.3]	na
UPDRS score	na	43 ± 19.2 [36.6;49.4]	na

Controls n = 24; all PD patients n = 41. The data are presented as mean ± standard deviation. The lower and upper 95% confidence limits are presented in brackets.

Abbreviations: EEG, electroencephalogram; L‐DT, laser detection threshold; L‐PT, laser pain threshold; LEP, laser‐evoked potential; LEP‐ HQ, laser evoked potential habituation quotient; LP‐HQ, laser pain habituation quotient; na, not applicable; ns, not significant; N2P2‐1, N2P2 potential of stimulation block 1; N2P2‐2, mean N2P2 potential of stimulation block 2 etc.; NRS, numeric rating scale (0 no pain to 10 worst imaginable pain); N2‐1, mean N2‐ latency of stimulation block 1; N2‐2, mean N2‐ latency of stimulation block etc.; Pain‐1, mean pain rating of stimulation block 1; Pain‐2, mean pain rating of stimulation block 2 etc,; PDQ, pain DETECT questionnaire; P2‐1, mean P2‐ latency of stimulation block 1; P2‐1, mean P2‐ latency of stimulation block 2 qtc.; UPDRS, Unified Parkinson's Disease Rating Scale.

**TABLE 2:** Demographic data, EEG, and pain values as well as PDQ score, UPDRS, and QST data for patients with and without pain

	Painful	Painless	*P*‐value
Age (y)	59.6 ± 9.3 [55.2;63.9]	64 ± 10 [59.5;68.6]	ns
Disease duration (y)	7.7 ± 4.1 [5.8;9.6]	10.3 ± 4.5 [8.2;12.3]	ns
Height (cm)	172.8 ± 10.3 [168;177.6]	173.7 ± 9.4 [169.3;178]	ns
L‐DT	1.3 ± 0.3 [1.1;1.5]	1.1 ± 0.6 [0.8;1.4]	ns
L‐PT	2.5 ± 0.4 [2.2;2.7]	2.3 ± 0.7 [1.9;2.6]	ns
N2P2‐1 (μV)	14.5 ± 9 [9.1;19.9]	9.5 ± 7.5 [5.7;13.4]	ns
N2P2‐2 (μV)	14.7 ± 10.1 [8.6;20.8]	8 ± 6.8 [4.5;11.4]	0.009
N2P2‐3 (μV)	14.6 ± 15.2 [5.4;23.8]	7.5 ± 6.2 [4.3;10.7]	ns
N2P2‐4 (μV)	13.4 ± 10.8 [6.8;19.9]	8.2 ± 6.4 [4.9;11.5]	ns
LEP‐HQ	1 ± 0.6 [0.7;1.4]	1 ± 0.5 [0.7;1.3]	ns
Pain‐1 (NRS)	4.9 ± 2 [3.9;5.8]	5 ± 2.4 [3.8;6.2]	ns
Pain‐2 (NRS)	4.1 ± 1.8 [3.3;5]	4.1 ± 2.1 [3.1;5.1]	ns
Pain‐3 (NRS)	4.3 ± 1.9 [3.4;5.2]	3.8 ± 1.9 [2.9;4.8]	ns
Pain‐4 (NRS)	4 ± 1.9 [3.1;4.9]	3.4 ± 1.9 [2.5;4.4]	ns
LP‐HQ	0.9 ± 0.3 [0.7;1]	0.8 ± 0.3 [0.6;0.9]	ns
N2‐1 (ms)	236.1 ± 34 [215.5;256.6]	250.3 ± 23.8 [237.6;262.9]	ns
N2‐2 (ms)	242.8 ± 39 [219.2;266.3]	250.1 ± 25 [236.8;263.4]	ns
N2‐3 (ms)	231.6 ± 40 [207.5;255.8]	254.1 ± 27 [239.7;268.5]	0.03
N2‐4 (ms)	235.3 ± 35.7 [213.7;256.9]	249.6 ± 25.7 [236;263.3]	ns
P2‐1 (ms)	328.2 ± 47.3 [299.6;356.7]	329.1 ± 35.8 [310.1;348.2]	ns
P2‐2 (ms)	326.5 ± 42.5 [300.8;352.2]	316.9 ± 35.2 [298.1;335.6]	ns
P2‐3 (ms)	332 ± 51.9 [300.7;363.4]	324.9 ± 26.2 [310.9;338.9]	ns
P2‐4 (ms)	332.2 ± 44.7 [305.2;359.2]	323.8 ± 28.4 [308.7;338.9]	ns
PDQ score	9.9 ± 6.8 [6.7;13]	2.1 ± 4.1 [0.1;4]	<0.001
UPDRS score	45.9 ± 20.4 [36.1;55.7]	39.9 ± 18 [31;48.8]	ns
DMA	1.5 ± 6.7 [−1.6;4.6]	2.4 ± 5.9 [−0.6;5.4]	ns
CDT *z* score	−0.7 ± 1.3 [−1.3;‐0.1]	−0.8 ± 1.3 [−1.4;‐0.1]	ns
WDT *z* score	−0.8 ± 1.3 [−1.5;‐0.2]	−1.3 ± 1.6 [−2.2;‐0.5]	ns
TSL *z* score	−1.1 ± 1.3 [−1.6;‐0.5]	−1.4 ± 1.2 [−2;−0.8]	ns
CPT *z* score	0.8 ± 1.2 [0.3;1.4]	1.2 ± 1.3 [0.5;1.8]	ns
HPT *z* score	1 ± 2 [0.1;1.9]	0.3 ± 1.9 [−0.7;1.2]	ns
MDT *z* score	–0.8 ± 2.1 [−1.8;0.2]	−0.9 ± 1.8 [−1.8;0]	ns
MPT *z* score	1.3 ± 1.1 [0.8;1.8]	1.2 ± 1.5 [0.4;1.9]	ns
MPS *z* score	1.4 ± 1.4 [0.8;2.1]	1.7 ± 1.5 [0.9;2.4]	ns
WUR *z* score	−0.5 ± 0.8 [−0.8;‐0.1]	−0.2 ± 0.9 [−0.6;0.2]	ns
VDT *z* score	−0.4 ± 1.7 [−1.2;0.4]	−1.1 ± 1.8 [−2;−0.3]	ns
PPT *z* score	–0.3 ± 1.7 [−1.1;0.5]	−1.2 ± 1.3 [−1.8;‐0.6]	ns

Painful: patients with current pain ≥1, n = 20; painless: current pain 0, n = 21. The data are presented as mean ± standard deviation. The lower and upper 95% confidence limits are presented in brackets.

Abbreviations: EEG, electroencephalogram; LEP, laser‐evoked potential; LEP‐HQ, laser evoked potential habituation quotient; LP‐HQ, laser pain habituation quotient; L‐DT, laser detection threshold; L‐Pain‐1, mean pain rating of stimulation block 1; na, not applicable; ns, not significant; N2P2‐1, N2P2 potential of stimulation block 1; N2P2‐2, mean N2P2 potential of stimulation block 2 etc.; NRS, numeric rating scale (0 no pain to 10 worst imaginable pain); N2‐1, mean N2‐ latency of stimulation block 1; N2‐2, mean N2‐ latency of stimulation block etc.; Pain‐2, mean pain rating of stimulation block 2 etc,; PDQ, pain DETECT questionnaire; PPT, pressure pain threshold; PT, laser pain threshold; P2‐1, mean P2‐ latency of stimulation block 1; P2‐1, mean P2‐ latency of stimulation block 2 qtc.; QST, quantitative sensory testing; UPDRS, Unified Parkinson's Disease Rating Scale.

**FIG. 2:**
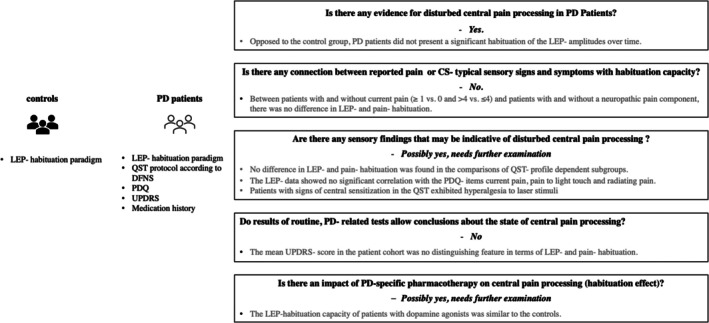
Overview of research questions and findings. DFNS, German Research Network of Neuropathic Pain; LEP, laser‐evoked pain; PD, Parkinson's disease; QST, quantitative sensory testing; UPDRS, Unified Parkinson's Disease Rating Scale.

For an overview of the conducted analyses, see Fig. 2. The QST profiles of the patients are shown in Figure S1 (entire cohort) and Figure S2 (divided by patients with and without current pain).

### Evidence for Disturbed Central Pain Processing in PD Patients

#### LEP‐Habituation Analyses

##### All PD Patients

**FIG. 3:**
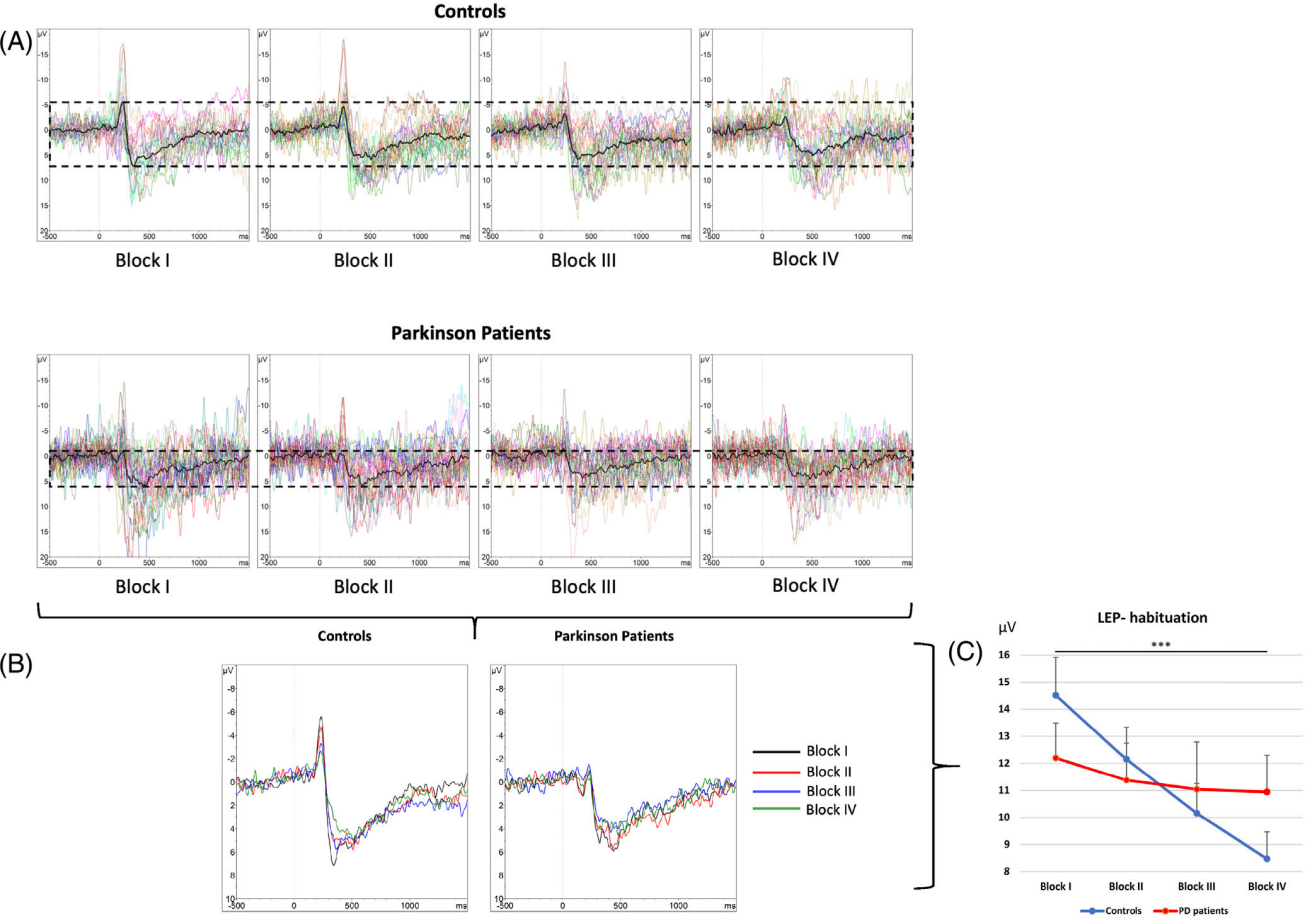
LEP grand averages of controls and PD patients: (**A**) block I–IV, (**B**) directly overlapped, (**C**) LEP amplitudes of controls and PD patients, block I–IV. LEP, laser‐evoked potential; PD, Parkinson's disease. Values presented with standard error.

Over the time course of four stimulation blocks, PD patients did not present a significant habituation of the N2P2 amplitudes (see Fig. 3A–C). The pain ratings, on the contrary, declined significantly (*F*_1.99;71.54_ = 12.7, *P* < 0.001; see Fig. S3). The LEP latencies, as expected, did not change over time.

##### Control Group

The control group exhibited a significant habituation effect both for N2P2 amplitudes (*F*_3;69_ = 19.9, *P* < 0.001; Fig. 3A–C) and for LPRs (*F*_1.88;52.58_ = 13.65, *P* < 0.001; Fig. S3). The LEP latencies did not change over time.

##### Control Group versus All PD Patients

In the group comparison, a significant difference between PD patients and controls in the time course of N2P2 amplitudes (*F*_1.88;97.32_ = 3.9, *P* = 0.026; see Fig. 3A–C) was found but no difference for the pain ratings over time.

#### Comparison of Group Variables

##### Control Group versus All PD Patients

A significant difference was found for the LEP perception (*P* = 0.003, patients > controls) and pain threshold (*P* = 0.04, patients > controls), the LEP‐habituation quotient (*P* = 0.003, patients > controls), LPRs in stimulation block I (*P* = 0.025, patients > controls), block II (*P* = 0.009, patients > controls), block III (*P* = 0.002, patients > controls), and block IV (*P* = 0.025, patients > controls).

### Is There Any Connection between Reported Pain or CS‐Typical Sensory Signs and Symptoms and Habituation Capacity?

There were no significant differences between patients with current pain ≥1 versus 0 and >4 versus ≤4, nor between patients with possible neuropathic pain (NP) component versus without (*P* > 0.05 for all comparisons; for more details see Data S2: Results).

#### Correlation of EEG Parameters with Current Pain, Pain to Light Touch, and Radiating Pain

The LP habituation coefficient showed a positive correlation with radiating pain (*P* = 0.031) but failed to reach statistical significance after correction for multiple testing.

None of the other correlation analyses were significant.

#### Comparison of Group Variables

##### PD Patients with Current Pain ≥1 versus 0

There was a significant difference in the PDQ sum score (*P* < 0.001, with current pain > without), the N2P2 amplitude of block II (*P* = 0.009, with current pain > without), and the N2 latency of block III (*P* = 0.032, with current pain < without).

##### PD Patients with Current Pain >4 versus ≤4

Therewas a significant difference in the PDQ sum score (*P* < 0.001, patients with current pain >4 vs. ≤4).

##### PD Patients with Possible NP Component versus without Pain

There was a significant difference in current pain (*P* = 0.007, with NP component > without), mechanical detection threshold (MDT) *z* score (*P* = 0.021, without NP component in loss range), and wind‐up ratio (WUR) *z* score (*P* = 0.024, without NP component more in loss range).

### Sensory Findings That May Be Indicative of Disturbed Central Pain Processing

#### LEP‐Habituation Analyses

When the time course of N2P2 amplitudes and pain ratings was analyzed, there were no significant differences between the QST‐item subgroups (ie, CDT loss vs. without, warm detection threshold (WDT) loss vs. without MDT loss vs. without, mechanical pain sensitivity (MPS) gain vs. without, mechanical pain threshold (MPT) gain vs. without, PPT loss vs. without).

#### Correlation of EEG Parameters with QST

LPRs of block I showed a positive correlation with the vibration detection threshold (VDT) *z* score (*P* = 0.027) but failed to reach statistical significance after correction for multiple testing.

No other correlation analyses were significant.

#### Comparison of Group Variables

##### PD Patients with CDT Loss versus without Loss

The CDT loss group (n = 9) presented significantly more loss of function within the parameters WDT (*P* = 0.002), thermal sensory limen (TSL) (*P* < 0.001), and PPT (*P* = 0.016).

##### PD Patients with WDT Loss versus without Loss

The WDT loss group (n = 10) was significantly older (*P* = 0.003) and presented significantly more loss of function within the parameters CDT (*P* = 0.037), TSL (*P* < 0.001), heat pain threshold (HPT) (*P* = 0.002), WUR (*P* = 0.032), and PPT (*P* = 0.034), whereas MPT (*P* = 0.018) and MPS (*P* = 0.032) were significantly more in the range of gain of function.

##### PD Patients with MDT Loss versus without Loss

The MDT loss group (n = 10) presented less pain (*P* = 0.022), a lower PDQ sum score (*P* = 0.009), a lower LEP perception threshold (*P* = 0.026), a lower LEP N2P2 habituation quotient (*P* = 0.017), a prolonged N2 latency within block I (*P* = 0.036), and P2 latency within block II (*P* = 0.044).

##### PD Patients with MPS Gain versus without Gain

Patients with MPS gain (n = 15) presented significantly lower LP thresholds (*P* = 0.009), higher dynamic mechanical allodynia (DMA) scores (*P* = 0.031), and lower TSL scores (*P* = 0.029).

##### PD Patients with MPT Gain versus without Gain

No significant group differences were found for patients with MPT gain (n = 12).

##### PD Patients with PPT Loss versus without Loss

A significant difference was detected in the LP threshold (*P* = 0.031, with PPT loss < without) and the CDT *z* score (*P* = 0.041, patients with PPT loss [n = 13] more on the loss range).

### Implications of Routine Clinical Tests in PD Patients for Habituation Capacity

#### LEP‐Habituation Analyses

When the time course of N2P2 amplitudes and LPRs was analyzed, there were no significant differences in the analyses looking into patients with a UPDRS score of >42.3 versus <42.3 and patients with agonist intake versus without.

#### Correlation of EEG Parameters with UPDRS

No significant correlations were found.

#### Comparison of Group Variables

There were no significant differences between patients with a mean UPDRS score of >42.3 versus <42.3.

### Impact of PD‐Specific Pharmacotherapy on Central Pain Processing

The mean l‐DOPA equivalent dosage was 934.4 ± 387.1 mg (lower control limit [LCL]: 812.2 mg and upper control limit [UCL]: 1056.6 mg).^72^, ^73^, ^74^ Five patients received l‐DOPA monotherapy. Two patients did not receive l‐DOPA (1 was treated with a dopamine agonist, the other with an agonist + Monoamine oxidase‐ B [MAO‐B] inhibitor). The remaining 34 patients received a combination of different PD drugs such as levodopa, dopamine agonists, Catechol‐O‐methyltransferase (COMT) inhibitors, MAO‐B inhibitors, or amantadine (Table S1).

#### LEP‐Habituation Analyses

We found significantly less LEP habituation in patients without agonists (patients: n = 7, controls: n = 24; *F*_3;87_ = 6.75, *P* < 0.001) and in patients with MAO‐B‐inhibitors (patients: n = 11, controls: n = 24; *P* < 0.01), with no other significant subgroup differences (detailed results are presented in Data S2: Results).

#### Correlation of EEG Parameters with l‐DOPA Equivalent Dose and Clinically Assessed Parameters

No correlation of the l‐DOPA equivalent dose with any of the assessed parameters (habituation quotient of LEP amplitudes and LPR, QST, PDQ, age, disease duration) was found (*P* > 0.1 for all comparisons).

#### Comparison of Group Variables

##### Patients with an Intake of ≥934 versus <934 mg Equivalent Dose of l‐DOPA

No significant differences were found between patients with an equivalent dose of ≥934 and <934 mg of l‐DOPA.

##### Patients with Agonists versus without Agonists

A significant difference was observed between these groups for the N2P2 amplitude in block I (*P* = 0.02, agonist > no agonist) and block II (*P* = 0.01, agonist > no agonist) and the MDT *z* score (*P* = 0.02, with agonists more in the loss area).

##### Patients with MAO‐B Inhibitors versus without Inhibitors

No significant differences were found.

## Discussion

The present study verifies the hypothesis of disturbed central pain processing in patients with PD even in pain‐free patients, shown by a lack of LEP‐habituation paradigm to a cohort of off‐state PD patients and thereby adding electrophysiological evidence to the discussion on PD‐related alterations in central pain processing.^27^, ^75^ The repetitive application of noxious laser stimuli will normally result in a decrease in N2P2 amplitude. A reduced LEP‐habituation capacity has been found in various chronic pain conditions and ultimately interpreted as a sign of CS.^34^, ^35^, ^49^, ^52^, ^53^, ^76^

Several mechanisms are considered to be involved in the habituation effect,^34^, ^53^, ^67^, ^77^, ^78^, ^79^, ^80^, ^81^ which appears to be associated with the endogenous, antinociceptive system.^82^, ^83^ The ACC,^53^, ^84^ the primary and secondary somatosensory cortex, the insula, the prefrontal cortex,^85^, ^86^, ^87^ and the thalamic nuclei^88^ are part of a neuroanatomic overlap between pain‐processing structures and those involved in PD‐associated pathology (Fig. 1). Fittingly, previous research pointed to the role of dopaminergic neurotransmission in the habituation to painful stimuli.^87^ A functional impairment resulting from this overlap is hereby shown through our results.

A relevant value of this study is the broad characterization of the patient group allowing to examine possible connections between somatosensory, pharmacological, and clinical measures. We observed no correlation of LEP‐ or LPR‐habituation capacity with measures of pain, pharmacotherapy, or the UPDRS. This is in line with a previous study that examined CPM in patients with restless legs syndrome^89^ and postulated a defect in the endogenous inhibitory pain system, which may be the case in PD patients also. To further examine this question, future studies should include specific paradigms (ie, CPM^89^, ^90^, ^91^, ^92^) to assess the diffuse noxious inhibitory control of the brainstem that is involved in the descending pain modulation.^93^

The control group detected laser stimuli at lower energy densities than the patients, which reflects heat pain hypoalgesia and may indicate small‐fiber function loss (discussed later^94^, ^95^). Possibly associated with CS processes, PD patients exhibited significantly higher pain ratings, which has previously been discussed to be dopamine related.^7^, ^25^, ^75^, ^96^, ^97^

Having found electrophysiological signs of CS in PD patients, one question was whether and how LEP parameters correspond with reported pain, using the PDQ item for current pain from two subgroups: patients with and without pain.

We found no differences between patients with and without pain for LEP and LP habituation. A previous QST study reported no difference between painful and painless neuropathies for small‐fiber function.^98^ An abnormal nociceptive processing in pain‐free PD patients has previously been discussed.^27^, ^47^ The hereby reported lack of LEP habituation in PD patients regardless of pain (1) supports the assumption that dopamine plays a role in pain modulation (for review, see Viseux and colleagues^6^) and (2) provides electrophysiological evidence that PD‐related pathophysiological changes occur on a central level and most likely involve CS mechanisms.

Another major point of interest was whether any sensory findings assessed using QST would point to a reduced capacity for habituation as CS proxy. Peripheral neuropathy is frequent in PD patients,^99^, ^100^, ^101^, ^102^, ^103^, ^104^ with negative effects on postural stability and sensory perception.^105^ As an integral part of the diagnostic workup of NP,^106^, ^107^ QST has contributed to this finding.^88^, ^94^, ^97^, ^108^, ^109^, ^110^, ^111^, ^112^, ^113^ Among our off‐state patients whose EEG data did not comply with our inclusion criteria (which resulted in EEG data loss), a large proportion exhibited a cold detection threshold (CDT) loss and an NP component according to the PDQ, presumably caused by peripheral neuropathy. Particularly, peripheral neuropathy can be a direct consequence of PD itself, with deafferentation and deposition of α‐synuclein in small fibers.^94^, ^114^, ^115^ A small‐fiber loss could be observed in a significant number of our patients (eg, CDT loss in 9/WDT loss in 10). Patients with MPS gain interestingly perceived laser stimuli as painful at a lower laser energy density than those without, exhibiting a hyperalgesia that may be reflective of CS. MPS has been proposed as a valuable marker for CS in a previous work.^64^ Patients with MDT loss (representative of Aß‐function loss) had significantly lower ratings for current pain and a lower PDQ sum score than those without. They also offered a higher LEP perception threshold and interestingly exhibited a stronger habituation effect than patients without MDT loss (lower N2P2 habituation quotient), which may be explained as follows: a degeneration of peripheral fibers with loss of function will lead to reduced activity in the spinal cord and thus less pain and sensitization. To be able to perceive pain, the structures conveying the sensation must be intact.^116^

Our results are congruent with previous findings on evidence for a heightened sensitivity toward noxious stimulation, for both thermal and mechanical stimuli.^97^, ^117^, ^118^, ^119^, ^120^

Concerning implications of routine clinical tests in PD patients and the impact of PD‐specific pharmacotherapy on central pain processing, we found the UPDRS did not show any significant difference (neither for LEP and LP habituation nor for other parameters). Furthermore, the EEG data showed no association with the UPDRS. Thus, in our cohort, the routine clinical tests conducted in PD patients ahead of DBS did not provide information on the question of the presence of a dysfunctional central pain processing (ie, reduced habituation capacity).

In this study, we found no association between the mean l‐DOPA equivalent dose and the EEG data, pain, and the clinically assessed parameters during the off‐ state. The analyses would have to be repeated in the on‐state to examine the direct effect of l‐DOPA on sensory thresholds, but this was not the scope of this study. Currently, it remains unclear whether dopamine possesses genuine antinociceptive qualities or works as a modulatory agent.^112^ For instance, some authors reported an increase in pain thresholds in PD patients for cold pain and heat pain through l‐DOPA treatment.^75^, ^121^ Others reported no effect of l‐DOPA on thermal and mechanical pain thresholds whatsoever.^110^ Due to the lack of impact of apomorphine on pain thresholds, it is presumed that the modulatory effect of dopamine on pain may in fact be conveyed through monoamine pathways (through conversion into noradrenaline and serotonin).^122^

Agonists and MAO‐B inhibitors have also been subject to studies assessing pain outcomes in PD patients (for review, see Viseux and colleagues^6^). Both rotigotine and safinamide (not indicated for treating PD‐induced pain) have been found to have favorable effects on pain.^123^, ^124^, ^125^, ^126^, ^127^, ^128^

Interestingly, our comparison of LEP and LP habituation between patients with and without agonists allows to carefully consider whether agonists influence CS processes: patients without agonists exhibited a significantly reduced LEP habituation compared to controls, whereas this observation could not be made for comparing controls and patients with agonists. These results must be interpreted with caution, and further studies are necessary as there were only 7 patients without agonists whose EEG data complied with our inclusion criteria. This observation is especially intriguing, as recent discussions turned to sensitization at cortical levels to explain the comorbidity of chronic pain with anxiety and depression.^4^, ^129^ The prevention thereof (perhaps through agonists, according to our data) may be beneficial for both target symptoms: pain and mood.

## Limitations

Our approach to subgroup analyses deviates from the standard recommendation of classifying patients into specific pain categories. Although we support this method, our sample size, though relatively large for LEP studies, was insufficient for meaningful group comparisons based on pain phenotypes. Particularly, only 4 patients met the criteria for “probable” NP (PDQ ≥19), 1 of them having EEG data meeting inclusion criteria. Moreover, classifying pain as “definitely” neuropathic in PD is challenging (see Ciampi De Andrade et al^4^), and PD patients often experience multiple pain types simultaneously.^2^, ^7^

The absence of EEG correlations with QST measures such as MPS may stem from these factors, emphasizing the need for larger cohorts to enable mechanistic subgroup analyses (eg, via QST clusters), which is crucial for advancing individualized pain treatment in PD.

Finally, some findings remain limited due to the small sample size and EEG segment loss, with LEP disruptions being inherent to peripheral neuropathy and further exacerbated by the off‐state during examination.

## Conclusion

We hereby report electrophysiological evidence for an impaired central pain modulation in PD patients regardless of pain presentation and individual sensation. Our results verify previous research, indicating a substantial overlap between the neuroanatomic structures involved in pain processing and PD pathology. We suggest a predisposition to chronic pain due to a lack of LEP habituation in PD patients thought to be caused by CS processes. This finding is observed even in pain‐free PD patients, suggesting that abnormal pain modulation is associated with or caused by dopamine deficiency. We examined the influence of pharmacotherapy and found no direct correlation between l‐DOPA equivalent dosage and pain processing.

## Author Roles

(1) Research Project: A. Conception, B. Organization, C. Execution; (2) Statistical Analysis: A. Design, B. Execution, C. Review and Critique; (3) Manuscript Preparation: A. Writing of the First Draft, B. Review and Critique.

D.K.: 1B, 1C, 2A, 2B, 2C, 3A, 3B.

J.L.: 1C, 2C, 3A, 3B.

J.F.: 2B, 2C, 3B.

M.S.: 1C, 3B.

S.‐C.F.: 1C, 3B.

S.N.: 1C, 3B.

J.S.: 1C, 3B.

S.P.: 1C, 3B.

D.B.: 1A, 1B, 2C, 3B.

R.B.: 1A, 1B, 2A, 2C, 3B.

P.H.: 1A, 1B, 1C, 2A, 2B, 2C, 3A, 3B.

## Supporting information


**Figure S1.** QST profile of all PD (Parkinson's disease) patients combined. The gray bar presents the range of normative data according to the DFNS database. Values outside the gray bar are considered abnormal. DFNS, German Research Network of Neuropathic Pain; QST, quantitative sensory testing.

**Figure S2.** QST profiles of PD (Parkinson's disease) patients with and without current pain. Gray bar: range of normative data according to the DFNS database. Values outside are considered abnormal. DFNS, German Research Network of Neuropathic Pain; QST, quantitative sensory testing.

**Figure S3.** Laser pain ratings of controls and PD patients, block I to IV. LP, laser pain; n.s., not significant; PD, Parkinson's disease. Values are presented with standard error.

**Data S1.** Methods.

**Data S2.** Results.

**Data S3.** Abbreviations.

**Table S1.** Pharmacotherapy of patients.

## Data Availability

The data that support the findings of this study are available from the corresponding author upon reasonable request.
