# Supplementary figures and images for: Electrophysiological Evidence for Impaired Central Pain Modulation in Parkinson's Disease
Source: Mov Disord. 2025 Aug 23;40(11):2393–406. doi: 10.1002/mds.70004 (PMC12661641; doi:10.1002/mds.70004)

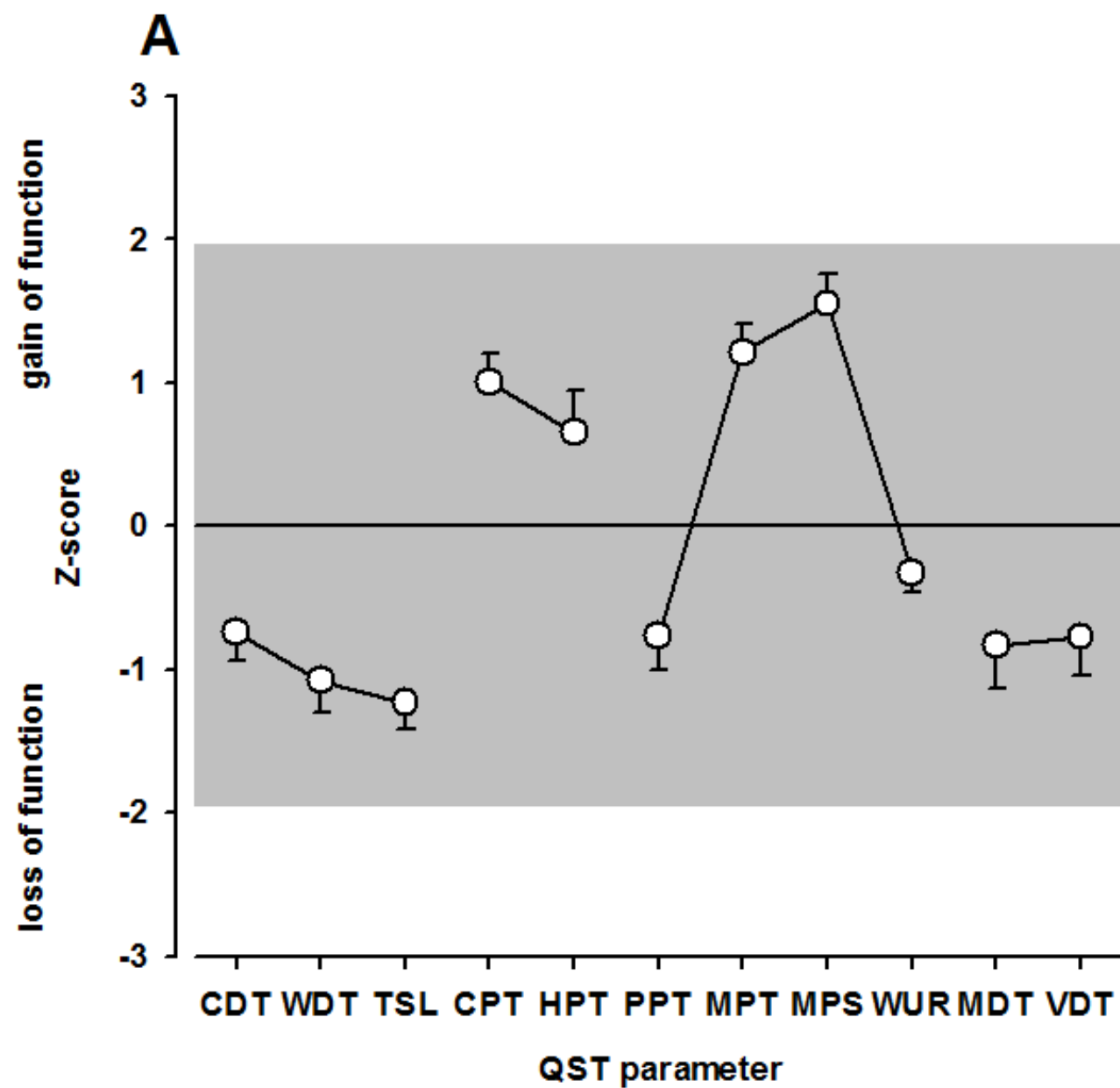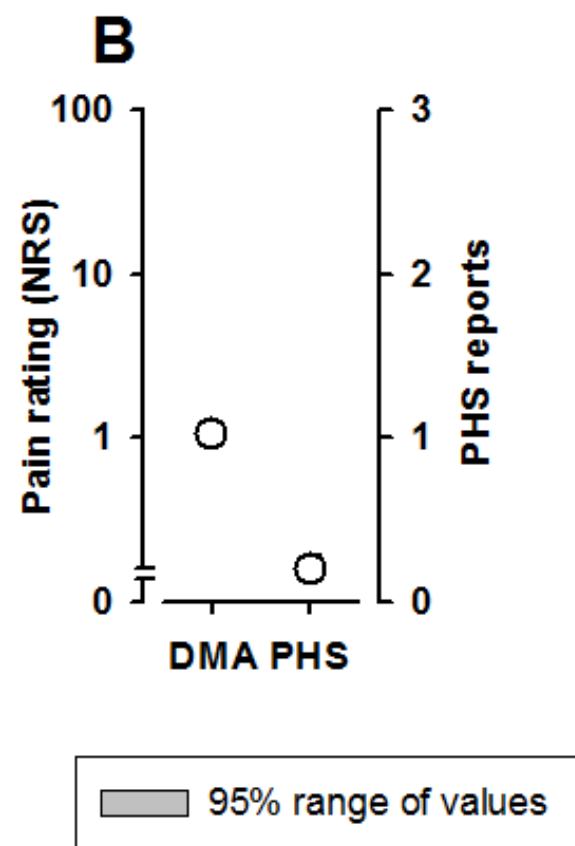

Supplement: Supplementary file 1 — Figure S1. QST profile of all PD (Parkinson's disease) patients combined. The gray bar presents the range of normative data according to the DFNS database. Values outside the gray bar are considered abnormal. DFNS, German Research Network of Neuropathic Pain; QST, quantitative sensory testing. [file MDS-40-2393-s003.pdf]

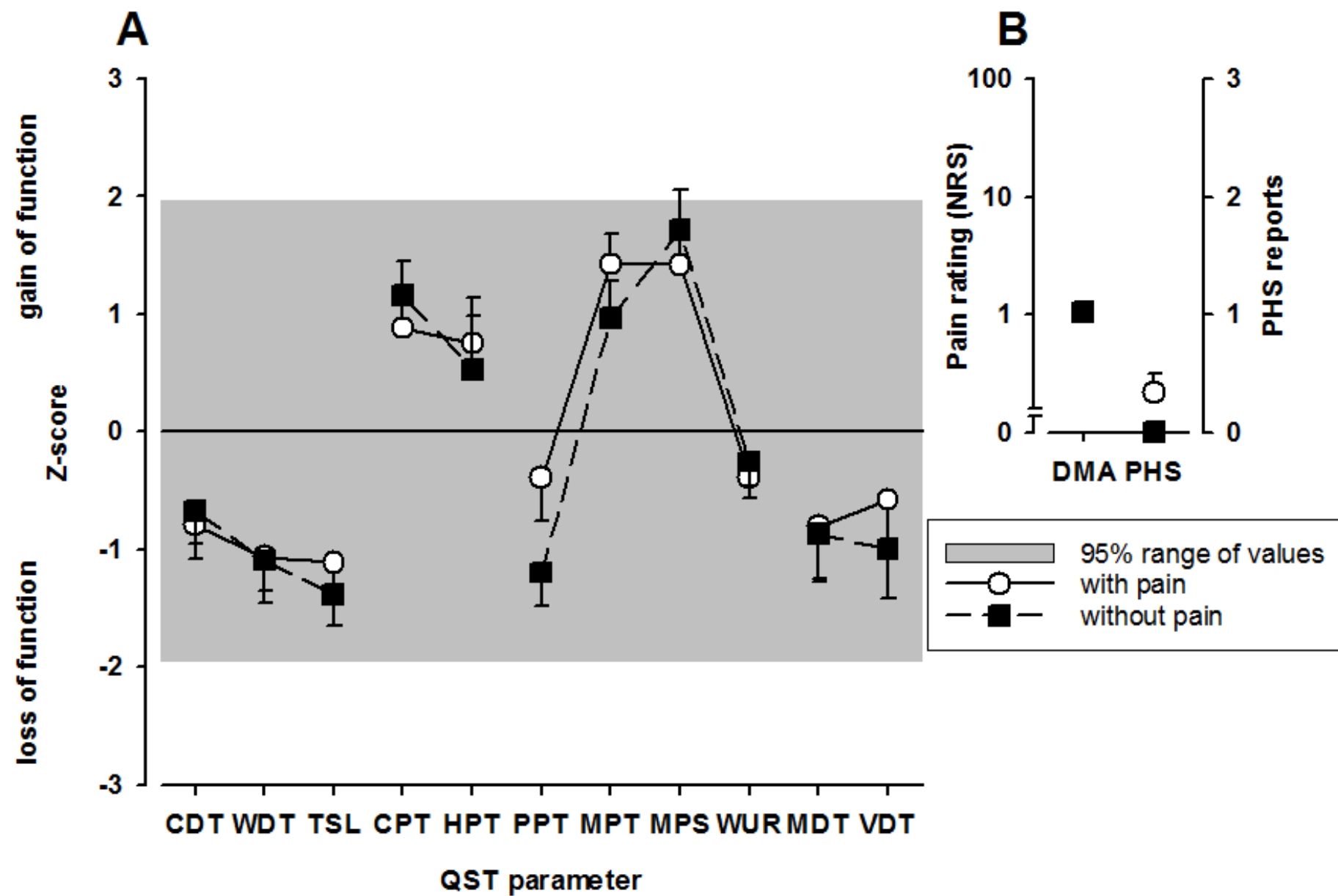

Supplement: Supplementary file 2 — Figure S2. QST profiles of PD (Parkinson's disease) patients with and without current pain. Gray bar: range of normative data according to the DFNS database. Values outside are considered abnormal. DFNS, German Research Network of Neuropathic Pain; QST, quantitative sensory testing. [file MDS-40-2393-s007.pdf]

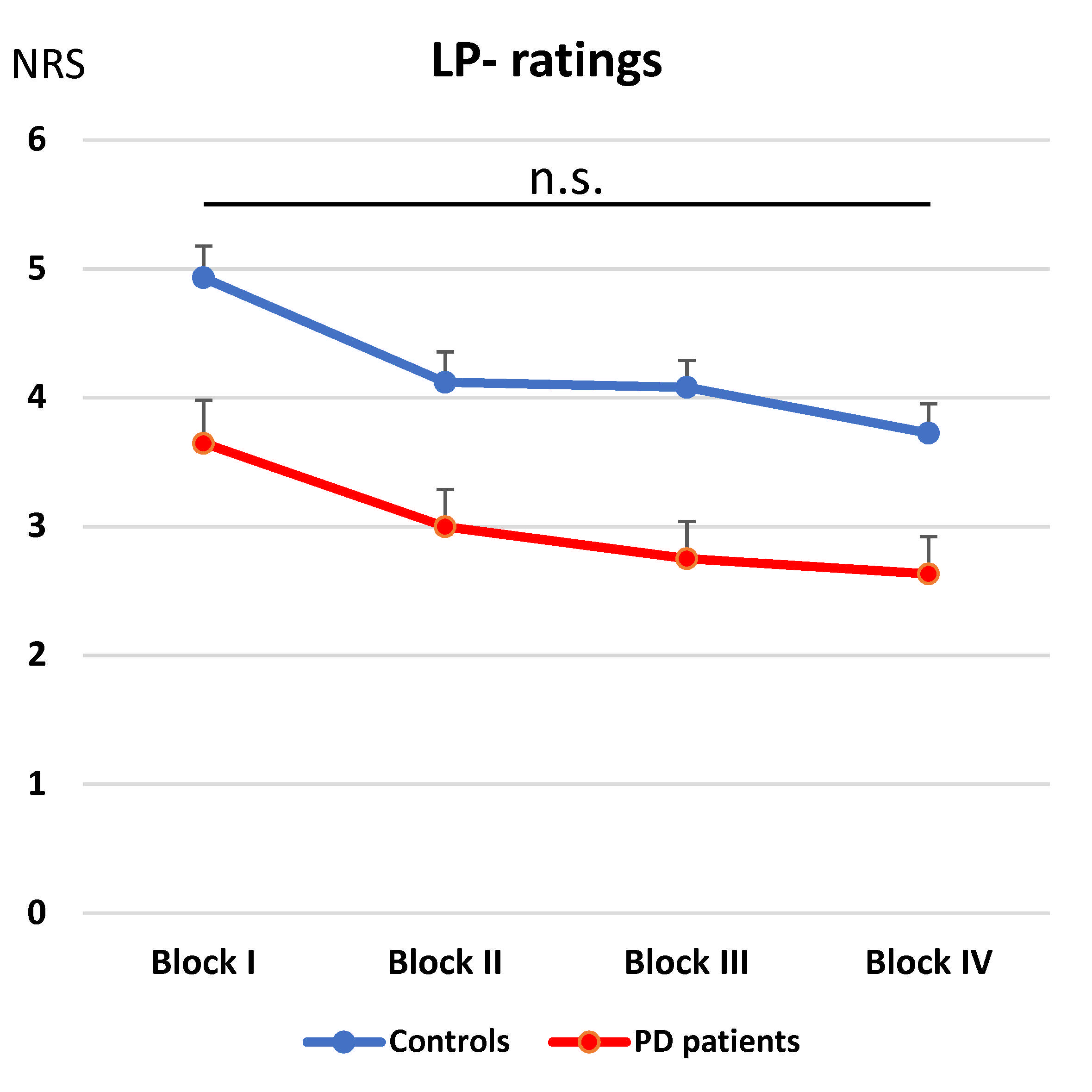

Supplement: Supplementary file 3 — Figure S3. Laser pain ratings of controls and PD patients, block I to IV. LP, laser pain; n.s., not significant; PD, Parkinson's disease. Values are presented with standard error. [file MDS-40-2393-s002.tif]
