# Supplementary material for: Electrophysiological Evidence for Impaired Central Pain Modulation in Parkinson's Disease
Source: Mov Disord. 2025 Aug 23;40(11):2393–406. doi: 10.1002/mds.70004 (PMC12661641; doi:10.1002/mds.70004)
Supplement: Supplementary file 4 — Data S1. Methods. [file MDS-40-2393-s004.docx]

**Supplements 1 - Methods**

**General information**

General inclusion and eligibility criteria were sufficient language skills, being of legal age for individual consent for research participation (18 years or older) and being diagnosed with PD (Hoehn & Yahr stage 2 or 3 ^54^). Exclusion criteria for patients were symptomatic parkinsonism, atypical parkinsonism, other neurological diseases associated with altered sensory perception and chronic pain diseases, current or previous drug or alcohol abuse, breastfeeding or pregnant women. For the control group, exclusion criteria were a chronic pain condition or psychiatric conditions such as depression and ongoing therapy with potent analgesics (WHO II). After the assessment of general patient history, all individuals underwent a neurological exam by a qualified physician.

### painDETECT Questionnaire (PDQ)

The PDQ has been developed as a screening questionnaire for the detection of a NP component in patients witch chronic pain ^57^. Although not validated for the assessment of CS, the PDQ was associated with the Central Sensitization Inventory (CSI) score ^58^ and has been reported to be able to assess aspects of CS ^59,60,61^ which is why we considered it suitable for this study. The assessed domains are general pain intensity, the course and distribution of pain, and seven sensory signs (burning, tingling/ prickling, painful to light touch, sudden pain attacks, painful to cold or heat, numbness, pressure pain). The sensory signs are rated on a six-point Likert scale (0 = never, 5 = very strongly). All components will add up to a sum score from 0 to 38, a result of ≥19 points indicating a >90% probability that a NP component is present while ≤12 points make it unlikely .

## QST

Somatosensory testing was realized with QST according to the German Research Network on Neuropathic Pain (DFNS)^55,56^. Z-scores outside the limits of a 95%-confidence interval of healthy controls of the DFNS database were defined as abnormal. Hence, Z- scores > +1.96 were interpreted as “gain” and < -1.96 as “loss” of function. Thermal testing was conducted with the TSA 2001-II (Medoc, Israel). Cold stimuli were applied with a ramp of 1°C/s reaching a minimal temperature of 0°C, warm stimuli. The location site for all individuals was the right dorsum of the hand.

## LEP recording and analysis

The entire setup for LEP – recording and documentation of pain ratings as well as the LEP- habituation paradigm have been conducted according to previously published protocols of our group ^35,51,67^. In a room with a constant temperature, individuals were comfortably positioned on a stretcher. According to the international 10–20 system, the following EEG electrodes were attached: Fz, Cz, PZ, C3, C4 with linked earlobes as reference for the recording of the N2/P2 component; T3 and T4 with Fz as reference ^51^. For artifact detection and grounding, an EOG and a wrist band were added. The testing site was the right-hand dorsum in every individual as previous LEP studies reported abnormalities regardless of the clinical affected side and habituation has been shown to occur bilaterally ^25, 48, 67^.

“A Nd:YAP 1340 Stimul Laser (neodymium:yttrium-aluminiumperovskite, DEKA Lasertechnologie GmbH, Mainburg, Germany) with a beam diameter of 5 mm and a stimulus duration of 5 ms was used. The individual’s detection threshold was determined by up-regulating the energy stepwise (beginning with 0.5 Joule and then stepwise by 0.5-Joule increases) until any sensation was felt. Beginning from the detection threshold, the energy was increased further until the individuals reported a distinct pinprick pain sensation between 3 and 6 on the numerical pain rating scale, which should be equal to a twofold detection threshold of the laser energy density”^35^.

This energy level was then defined as the laser pain threshold and used for the further testing protocol. The individuals were instructed not to blink and move their eyes while the stimulus was being applied and for 3 s until a ping tone emerged. With the ping tone, blinking was allowed, and the individuals were asked to rate the perceived laser pain intensity on a numerical rating scale (0 = no pain, 10 = most imaginable pain). For the LEP-habituation protocol, a total number of 100 stimuli were applied in 4 blocks à 25 stimuli. “To avoid receptor fatigue^69^ or sensitization^70^, the hand piece of the laser stimulator was moved slightly within the testing area. Between each laser stimulus, 8–12 s elapsed.” ^35^. Approximately 1 minute passed in between the stimulation blocks with a total protocol duration of approximately 23 minutes.

“The EEG was recorded with Brain vision recorder 1.2 using the BrainAmp MR plus EEG amplifier (Brain products GmbH, Gilching Germany) and analyzed with Brain Vision Analyzer 2.0 (Brain Products GmbH; Gilching, Germany, Version 2.0.3.6367). All frames which contained artifacts 0.5 s before the laser stimulus and 2 s afterwards due to movement or blinking were excluded from analysis during visual inspection. The EEG was band-pass filtered with 0.3–35 Hz; the sampling rate was 1000 Hz. The N2/P2 amplitude was measured from the most negative to the most positive peak. The N1 amplitude was measured from baseline to the N1 peak (baseline correction was performed using a  500 ms to 0 ms pre-stimulus interval). The latency of each Adelta component was measured from the stimulus onset (0 ms) to the peak of the averaged potentials (N2 latency)”^25,35,51^.

For our research questions, the N1 latency was irrelevant and was thus not included in the statistical analyses.

## Unified Parkinson’s Disease Rating Scale (UPDRS)

The MDS-UPDRS was developed to evaluate various aspects of Parkinson’s disease including non-motor and motor experiences of daily living and motor complications^71^. The score for the clinician scored motor evaluation, UPDRS-III, has been assessed in the OFF- stage as a routine diagnostic step ahead of DBS and its score included in this study.

## Statistical Analysis

Descriptive statistics were presented as mean, standard deviation, and lower and upper limits of a 95% confidence interval. Figures were prepared using means and standard error of the mean.

To account for research questions 2 - 5, PD patients have been divided into below- listed subgroups. Comparisons have been made in terms of LEP- and Laser pain- habituation (ANOVA, see below) as well as group variables (MWU, see below).

The following PD subgroups have been compared to each other:

- Clinical presentation with pain:

patients with vs. without current pain/ patients with current pain >4 vs. ≤4 as assessed through PDQ/ patients with possible NP component (i.e., PDQ score ≥12) vs. without (i.e., PDQ score <12)

- Loss of mall fiber function:

patients with CDT loss vs. without / patients with WDT loss vs. without

- Loss of large fiber function:

patients with MDT loss vs. without

- Central sensitization:

patients with MPS gain vs. without/ patients with MPT gain vs. without / patients with PPT loss vs. without

- Routine clinical PD test:

Patients with a UPDRS score > 42.3 vs. < 42.3 (42.3 being the mean UPDRS score of the inspected group)

- Pharmacotherapy:

Patients with agonist intake vs. without, patients with a mean equivalence dose of > 927 mg vs. < 927 L-Dopa (i.e., the mean dose of the inspected group), patients with MAO-B inhibitors vs. without.

### Habituation

Within one group (PD patients and healthy individuals each), the time course of the N2P2-amplitude as well as the subjective pain ratings (i.e., habituation effect) have been analyzed with a repeated measures ANOVA and post-hoc Bonferroni correction for pair-wise comparisons. For group comparisons of the habituation of N2P2- amplitudes and LPRs (i.e., PD patients vs. healthy individuals/ PD subgroup comparisons), a two-way ANOVA with time as within- and group as between-individuals factor was applied.

### Comparison of group variables

For the comparison of group variables (demographic data, QST parameters, N2P2- amplitudes, LPRs, N2- and P2- latencies, questionnaire results and the UPDRS score if applicable) among groups (PD patients vs. healthy individuals) and subgroups (see above), the Mann–Whitney U (MWU) test was used.

### Correlation analyses

To be able to include the habituation effect into the correlation analyses, a habituation coefficient has been formed for each patient by dividing the mean N2P2- amplitude/ the LPR of stimulation Block IV by the mean N2P2- amplitude/ the LPR Block I. A habituation coefficient near 1 indicates no or a very weak habituation effect a habituation coefficient of near 0, indicates a strong habituation effect.

Within all PD patients, the association between 5 LEP- parameters (i.e., N2P2- and pain- habituation quotient, N2P2 amplitude Block I, LPR Block I as well as N2 latency Block I) and the following parameters assessed in clinical routine were calculated:

- Current pain, PDQ item pain to light touch, PDQ item radiating pain (the last two being signs of CS [1–4])
- QST parameters (CDT, WDT, MDT, MPS, MPT, PPT, DMA)
- UPDRS score
- L-Dopa equivalence dose

To further inspect the effect of pharmacotherapy, the association between the L-DOPA equivalence dose and the above- mentioned EEG-parameters, QST parameters, PDQ sum score, age and disease duration has been calculated.

For the correlation analyses, to adjust the significance level (p < 0.05) for multiple testing, the p-values were multiplied with the number of analyzed items.
