# Supplementary material for: Electrophysiological Evidence for Impaired Central Pain Modulation in Parkinson's Disease
Source: Mov Disord. 2025 Aug 23;40(11):2393–406. doi: 10.1002/mds.70004 (PMC12661641; doi:10.1002/mds.70004)
Supplement: Supplementary file 5 — Data S2. Results. [file MDS-40-2393-s006.docx]

**Supplement 2 – results**

## Is there any connection between reported pain or CS- typical sensory signs and symptoms with habituation capacity?

### LEP habituation analyses

#### PD Patients with Current Pain ≥ 1 vs. 0

There were no significant differences for either the habituation of N2P2- amplitudes (pain n=19, no pain n=18) or LPRs (pain n=13, no pain n=17).

#### PD patients with current pain >4 vs. ≤4

There were no significant differences for either the habituation of N2P2- amplitudes (>4 n=6, ≤4 n=24). or LPRs (>4 n=9, ≤4 n=28).

#### PD patients with possible NP component vs. without

There were no significant differences for either the habituation of N2P2- amplitudes (possible NP n=4, NP unlikely n=26) or LPRs (possible NP n=7, NP unlikely n=30).

## Impact of PD-specific pharmacotherapy on central pain processing

### LEP habituation analyses

#### Control Group vs. PD Patients without Agonists

A significant difference between these groups in the development of N2P2- amplitudes (patients n=7, controls n=24; *F_3;87_ = 6.75;* p<0.001) was found. Interestingly, this was not the case for LPRs (patients n=10, controls n=24).

#### Control group vs. PD patients with MAO-B- inhibitors

A significant difference was found between these groups in the development of N2P2 amplitudes over time (p<0.01, patients n=11, controls n=24). Again, this was not the case for the LPRs.

#### Other subgroup analyses

In the analyses comparing the

- control group vs. patients with agonists,
- control group without MAO-B-inhibitors,
- PD patients with agonists vs. without
- PD patients with MAO-B- inhibitors vs. without,
- control group vs. patients with an equivalence dose of ≥ 934 mg L-Dopa and patients with an equivalence dose of < 934 mg L-Dopa, and finally
- PD patients with an equivalence dose of ≥ 934 mg vs. < 934 mg L-Dopa

there were no significant differences in the development of N2P2- amplitudes or LPRs over time.
