# Supplementary material for: Electrophysiological Evidence for Impaired Central Pain Modulation in Parkinson's Disease
Source: Mov Disord. 2025 Aug 23;40(11):2393–406. doi: 10.1002/mds.70004 (PMC12661641; doi:10.1002/mds.70004)
Supplement: Supplementary file 6 — Data S3. Abbreviations. [file MDS-40-2393-s001.docx]

**Abbreviations of QST parameters**

CDT = cold detection threshold

WDT = warm detection threshold

TSL = thermal sensory limen

CPT = cold pain threshold

HPT = heat pain threshold

PPT = pressure pain threshold,

MPT = mechanical pain threshold,

MPS = mechanical pain sensitivity

WUR = wind-up ratio

MDT = mechanical detection threshold

VDT = vibration detection threshold

DMA = dynamic mechanical allodynia

PHS = paradoxical heat sensation

**Abbreviations Table 1**

*L-DT= laser detection threshold, L-PT= laser pain threshold, N2P2-1= N2P2 potential of stimulation block 1, N2P2-2= mean N2P2 potential of stimulation block 2 etc., LEP- HQ= laser evoked potential habituation quotient, Pain-1= mean pain rating of stimulation block 1, Pain-2= mean pain rating of stimulation block 2 etc., NRS = numeric rating scale (0 no pain to 10 worst imaginable pain), LP-HQ= laser pain habituation quotient, N2-1= mean N2- latency of stimulation block 1, N2-2= mean N2- latency of stimulation block etc., P2-1= mean P2- latency of stimulation block 1, P2-1= mean P2- latency of stimulation block 2 qtc., PDQ= painDETECT questionnaire, UPDRS= Unified Parkinson’s Disease Rating Scale*

**Abbreviations Table 2**

*L-DT= laser detection threshold, L-PT= laser pain threshold, N2P2-1= N2P2 potential of stimulation block 1, N2P2-2= mean N2P2 potential of stimulation block 2 etc., LEP- HQ= laser evoked potential habituation quotient, Pain-1= mean pain rating of stimulation block 1, Pain-2= mean pain rating of stimulation block 2 etc., NRS = numeric rating scale (0 no pain to 10 worst imaginable pain), LP-HQ= laser pain habituation quotient, N2-1= mean N2- latency of stimulation block 1, N2-2= mean N2- latency of stimulation block etc., P2-1= mean P2- latency of stimulation block 1, P2-1= mean P2- latency of stimulation block 2 qtc., PDQ= painDETECT questionnaire, UPDRS= Unified Parkinson’s Disease Rating Scale. CDT = cold detection threshold, WDT = warm detection threshold, TSL = thermal sensory limen, CPT = cold pain threshold, HPT = heat pain threshold, PPT = pressure pain threshold, MPT = mechanical pain threshold, MPS = mechanical pain sensitivity, WUR = wind-up ratio, MDT = mechanical detection threshold, VDT = vibration detection threshold, DMA = dynamic mechanical allodynia.*
