# Supplementary material for: Electrophysiological Evidence for Impaired Central Pain Modulation in Parkinson's Disease
Source: Mov Disord. 2025 Aug 23;40(11):2393–406. doi: 10.1002/mds.70004 (PMC12661641; doi:10.1002/mds.70004)
Supplement: Supplementary file 7 — Table S1. Pharmacotherapy of patients. [file MDS-40-2393-s005.docx]

**Supplementary Table** Pharmacotherapy of patients

|  | Agonists | | | | COMT | | MAO-B | | NMDA | Levodopa | | total euqivalence dose without levodopa | total equivalence dose |
| --- | --- | --- | --- | --- | --- | --- | --- | --- | --- | --- | --- | --- | --- |
|  | Pramipexole | Piribedil | Ropinirole | Rotigotine | Entacapone | Opicapone | Rasagiline | Safinamide | Amantadine | Levodopa | depot |  |  |
| P01 |  |  |  |  | 1000 |  | 1 |  | 300 | 450 |  | 730.0 | 1180.0 |
| P02 | 1.05 |  |  |  |  | 50 |  |  |  | 500 |  | 216.0 | 716.0 |
| P03 | 3.3 | 50 |  |  |  | 50 |  |  |  | 300 |  | 587.4 | 887.4 |
| P04 | 2.1 |  |  |  | 1000 |  | 1 |  |  | 550 | 100 | 705.0 | 1355.0 |
| P05 | 3.15 |  |  |  | 1000 |  |  |  |  | 600 | 100 | 755.0 | 1455.0 |
| P06 |  | 200 |  |  |  |  |  |  |  | 400 |  | 200.0 | 600.0 |
| P07 | 3.5 |  |  |  | 1000 |  |  |  |  | 500 |  | 830.0 | 1330.0 |
| P08 | 0.7 |  |  |  | 1000 |  | 1 |  |  | 550 | 200 | 480.0 | 1230.0 |
| P09 | 2.1 |  |  |  | 1400 |  |  |  |  | 825 |  | 762.0 | 1587.0 |
| P10 |  | 150 |  |  |  |  | 1 |  |  |  |  | 250.0 | 250.0 |
| P11 |  |  |  | 8 |  |  |  |  |  |  |  | 240.0 | 240.0 |
| P12 |  |  | 4 |  |  |  |  |  |  | 300 | 100 | 55.0 | 455.0 |
| P13 |  |  |  |  |  | 50 |  |  | 200 | 500 |  | 266.0 | 766.0 |
| P14 |  |  |  |  |  |  |  |  |  | **1000** |  | **0.0** | **1000.0** |
| P15 | 2.62 |  |  |  |  |  |  |  |  | 400 | 100 | 349.3 | 849.3 |
| P16 |  |  |  |  | 800 |  | 1 |  |  | 600 | 100 | 339.0 | 1039.0 |
| P17 | 3.15 |  |  |  |  | 50 |  |  |  | 725 | 100 | 491.0 | 1316.0 |
| P18 | 1.05 |  |  |  | 1000 |  | 1 |  |  | 500 |  | 580.0 | 1080.0 |
| P19 |  |  | 8 |  |  |  |  |  |  | 600 | 100 | 135.0 | 835.0 |
| P20 |  |  | 4 |  |  | 50 |  |  |  | 300 |  | 146.0 | 446.0 |
| P21 |  |  |  | 8 |  |  |  |  | 200 | 400 |  | 440.0 | 840.0 |
| P22 |  |  |  |  |  |  |  |  |  | **300** |  | **0.0** | **300.0** |
| P23 |  |  |  |  |  |  |  |  |  | **300** |  | **0.0** | **300.0** |
| P24 | 0.52 |  |  |  |  |  | 1 |  | 200 | 200 | 100 | 349.3 | 649.3 |
| P25 | 1.57 |  |  |  | 1000 |  |  |  | 100 | 1000 |  | 654.3 | 1654.3 |
| P26 |  |  | 8 |  |  |  |  | 100 |  | 600 | 100 | 260.0 | 960.0 |
| P27 |  |  | 16 |  |  | 50 |  | 100 |  | 300 |  | 511.0 | 811.0 |
| P28 | 2.36 |  |  |  |  | 50 |  | 50 |  | 700 | 100 | 478.1 | 1278.1 |
| P29 | 2.1 |  |  |  | 600 |  | 1 |  |  | 300 |  | 598.0 | 898.0 |
| P30 |  |  | 24 |  |  |  | 1 |  |  | 400 | 100 | 555.0 | 1055.0 |
| P31 |  |  |  | 8 |  | 50 |  |  |  | 800 | 200 | 256.0 | 1256.0 |
| P32 |  |  |  |  |  | 50 | 1 |  |  | 500 |  | 166.0 | 666.0 |
| P33 |  |  | 3 |  | 800 |  |  |  |  | 700 |  | 324.0 | 1024.0 |
| P34 | 2.62 |  |  | 4 |  | 50 |  | 100 |  | 425 |  | 685.3 | 1110.3 |
| P35 |  |  |  | 8 |  | 50 |  |  | 200 | 1200 | 100 | 481.0 | 1781.0 |
| P36 |  |  | 6 |  |  | 50 |  | 100 | 400 | 400 |  | 711.0 | 1111.0 |
| P37 |  |  | 4 |  |  |  |  |  |  | 800 | 100 | 55.0 | 955.0 |
| P38 | 1.57 |  |  |  |  |  |  |  |  | 400 |  | 224.3 | 624.3 |
| P39 |  |  |  |  | 1200 |  | 1 | 50 |  | 550 | 100 | 571.0 | 1221.0 |
| P40 |  |  |  |  |  |  |  |  |  | **800** |  | **0.0** | **800.0** |
| P41 |  |  |  |  |  |  |  |  |  | **400** |  | **0.0** | **400.0** |
|  |  |  |  |  |  |  |  |  |  |  |  |  |  |
| *Agonists = dopamine agonists; COMT = COMT inhibitors; MAO-B = MAO-B inhibitors; NMDA = NMDA receptor antagonists; Levodopa = levodopa + benserazide; all dosages are given in mg. In bold black we indicated patients with levodopa mono therapy.*  *The equivalence dose was calculated according to*  *(1) Giossi R, Carrara F, Mazzari M, Lo Re F, Senatore M, Schicchi A, et al. Overall Efficacy and Safety of Safinamide in Parkinson's Disease: A Systematic Review and a Meta-analysis. Clin Drug Investig. 2021;41(4):321-39  (2) Tomlinson CL, Stowe R, Patel S, Rick C, Gray R, Clarke CE. Systematic review of levodopa dose equivalency reporting in Parkinson's disease. Mov Disord. 2010;25(15):2649-53and*  *(3) Schade S, Mollenhauer B, Trenkwalder C. Levodopa Equivalent Dose Conversion Factors: An Updated Proposal Including Opicapone and Safinamide. Mov Disord Clin Pract. 2020;7(3):343-5* | | | | | | | | | | | | | |
